# Enzymatic Hydrolysis Systems Enhance the Efficiency and Biological Properties of Hydrolysates from Frozen Fish Processing Co-Products

**DOI:** 10.3390/md23010014

**Published:** 2024-12-28

**Authors:** Maria Sapatinha, Carolina Camacho, Antónia Juliana Pais-Costa, Ana Luísa Fernando, António Marques, Carla Pires

**Affiliations:** 1 Department of Chemistry, Nova School of Science and Technology, Nova University Lisbon, Campus da Caparica, 2829-516 Caparica, Portugal; m.sapatinha@gmail.com; 2 Division of Aquaculture, Upgrading and Bioprospection, Portuguese Institute for the Sea and Atmosphere (IPMA, I.P.), Av. Doutor Alfredo Magalhães Ramalho 6, 1495-165 Algés, Portugal; ccamacho@ciimar.up.pt (C.C.); amarques@ipma.pt (A.M.); 3 Interdisciplinary Centre of Marine and Environmental Research (CIIMAR/CIMAR-LA), University of Porto, Terminal de Cruzeiros do Porto de Leixões, Av. General Norton de Matos s/n, 4450-208 Matosinhos, Portugal; julianapaiscosta@gmail.com; 4 MEtRICs, Department of Chemistry, Nova School of Science and Technology, Nova University Lisbon, Campus da Caparica, 2829-516 Caparica, Portugal; ala@fct.unl.pt

**Keywords:** bioactive compounds, Alcalase, Protana, sequential hydrolysis, simultaneous hydrolysis, anti-hypertensive, antioxidant, molecular weight distribution, zero waste

## Abstract

Co-products from the frozen fish processing industry often lead to financial losses. Therefore, it is essential to transform these co-products into profitable goods. This study explores the production of fish protein hydrolysates (FPH) from three co-products: the heads and bones of black scabbardfish (*Aphanopus carbo*), the carcasses of gilthead seabream (*Sparus aurata*), and the trimmings of Nile perch (*Lates niloticus*). Four enzymatic hydrolysis systems were tested: an endopeptidase (Alcalase, A), an exopeptidase (Protana, P), two-stage hydrolysis with an endopeptidase followed by an exopeptidase (A + P), and a single stage with endo- and exopeptidase (AP). The results show that combined enzymatic treatments, especially single-stage Alcalase and Protana (AP), achieved high protein yields (80%) and enhanced degrees of hydrolysis (34 to 49%), producing peptides with lower molecular weights. FPH exhibited significant antioxidant activity, in 2,2′-azino-bis(3-ethylbenzothiazoline-6-sulfonic acid) (ABTS) assays, with EC_50_ values below 5 mg/mL. Additionally, AP hydrolysates demonstrated over 60% angiotensin-converting enzyme (ACE) inhibition at 5 mg/mL, indicating potential antihypertensive applications. Antidiabetic and anti-Alzheimer activities were present, but at relatively low levels. AP hydrolysates, especially from gilthead seabream, proved to be the most promising. This study highlights the value of fish co-products as sources of functional peptides, contributing to waste reduction, and their potential applications in food, agriculture, and nutraceuticals.

## 1. Introduction

As 2030 approaches, concerns arise about the successful accomplishment of Sustainable Development Goals (SDGs). Currently, the world is facing a severe crisis, with over 40% of the global population experiencing food insecurity and uncertainty. Conflicts, extreme weather events, environmental degradation, and economic shocks are worsening this situation. Rising food costs further reveal the fragility of existing agri-food systems, emphasizing their vulnerability to climate variability [1]. As the global population grows rapidly, and is expected to reach 9.8 billion by 2050, a surplus pressure is being placed on food production systems and resource management. Therefore, meeting the rising global demand for protein is crucial, especially for maintaining healthy diets for children and the aging population [2]. The increasing demand for protein, particularly from fish, has led to a surge in fish processing activities that are expected to further increase, as fisheries and aquaculture production are projected to reach 205 Mt by 2032, with most of this increase coming from aquaculture with 100 Mt [1].

While fish may be consumed fresh, the majority is processed, leading to a substantial increase in the co-products generated. Fish processing industries (FPI) are significant contributors to food loss and waste (FLW), generating substantial amounts (20–80% of the fish weight) of biowaste in the form of heads, viscera, frames, and trimmings [3,4,5]. Traditionally used for animal feed or fish meal, co-product generators often fail to capitalize on the full potential of their nutritional and economic value [3].

To achieve sustainability, an innovative and cost-effective approach to utilizing underutilized resources, such as fish processing co-products, are necessary. This involves boosting alternative protein production and ensuring consumer acceptance through effective communication [6]. Immediate policy changes are vital for securing affordable, nutritious, and sustainable protein sources for the future [2,6]. Although co-products are now classified as Category 3 materials under European Regulation (EC) No.1069/2009, they have often been overlooked in the past. However, they may hold significant economic and environmental value, due to their valuable nutrient content, especially proteins (10–20% of total protein in fish) [5,7,8]. Also, their valorization contributes to the reduction of food waste, aligning with SDG 12, particularly target 12.3, which focuses on halving per capita global food waste and reducing food losses along production and supply chains.

Fish protein hydrolysates (FPH) have emerged as a promising solution to address the challenges of transforming co-products into high-value products. Co-products having such high protein content (ca. 14%) enable their conversion into valuable products [8]. FPH are, by definition, the product of the cleavage of protein in fish into smaller peptides and amino acids, usually resulting in amorphous, hygroscopic powders with high protein content [9]. These hydrolysates can be obtained using various methods, including chemical hydrolysis (acid and alkaline), autolysis, microbial fermentation, and enzymatic hydrolysis [9].

Although chemical hydrolysis is often used due to its low cost, enzymatic hydrolysis is preferred for its specificity and potential to preserve its nutritional value [10]. This process involves the use of proteases, such as endopeptidases and exopeptidases, which are proteolytic enzymes able to break protein chains by targeting specific peptide bonds [11]. Depending on the specificity, yield, degree of hydrolysis (DH), and desired functional properties, the interior or side chains of proteins can be targeted [9,11]. Commonly used proteolytic enzymes include Alcalase, trypsin, chymotrypsin, and papain [9,12]. Combinations of endo- and exopeptidases are increasingly being employed to achieve specific hydrolysis outcomes [13,14,15].

Enzymatic hydrolysis of fish proteins using sequential combinations of endo- and exopeptidases is a well-established process in food science and biotechnology [16,17,18,19]. However, the simultaneous application of these enzymes has received limited attention, particularly regarding the optimization of protein hydrolysis efficiency over time. Recent studies have highlighted the potential of umami peptides, which are generated during extended hydrolysis processes, not only for enhancing flavor profiles, but also for providing significant biological activities, including antihypertensive, antioxidant, and hypolipidemic effects [20]. Moreover, marine organisms have emerged as promising bioresources for these peptides, offering sustainable solutions for the efficient utilization of low-value seafood products and processing by-products [20,21]. Despite growing interest in enzymatic hydrolysis, the application of Protana Prime, a commercially available exopeptidase, remains underexplored in the context of FPH [18]. Its potential to enhance bioactivity and improve debittering properties presents a significant yet untapped opportunity for protein processing [19]. Thus, by optimizing enzymatic hydrolysis through the simultaneous application of both endo- and exopeptidases, the valorization of undervalued fish processing co-products may be promising. Low-value co-products can be transformed into high-value functional ingredients, enriched with enhanced umami flavors, and characterized by maximum protein conversion efficiency, resulting in smaller peptides with desirable bioactive properties [19,20].

FPH exhibit a wide range of functional properties, including emulsifying, foaming, gelling, and water-holding capacity, which are essential for their application in food systems [11]. Moreover, these hydrolysates possess valuable biological activities, such as antioxidant, anti-inflammatory, antihypertensive, and antimicrobial properties, making them desirable ingredients for functional foods and nutraceuticals [8,21,22,23]. By utilizing fish processing co-products through FPH production, it is possible to tailor high-value products while contributing to sustainable resource utilization. Peptides have significant potential as effective ingredients in health-promoting products, offering innovative solutions for enhancing overall well-being [21].

Gilthead seabream (GB) is a highly consumed and produced fish in southern Europe, with aquaculture production reaching 334 kt in 2022 [24,25]. This species represents the growing impact of aquaculture on fish protein production. Black scabbardfish (BS), with 4.8 kt caught in 2022, highlights the importance of preserving the traditional fisheries heritage off the Portuguese coast. It is one of the oldest exploited fish species in the country, and holds cultural and economic importance [24,26]. Nile perch (NP), with a catch of 230 kt, presents unique challenges due to its size and the complex nature of its co-products, making it a valuable model for addressing the industrial-scale processing of by-products [24]. Three types of co-products were used in this study: black scabbardfish heads (*Aphanopus carbo*, Lowe, 1839), gilthead seabream carcasses (*Sparus aurata*, Linnaeus, 1758) from aquaculture, and Nile perch trimmings (*Lates niloticus*, Linnaeus, 1758). These co-products were intentionally selected to encompass a broad spectrum of production systems between traditional fisheries, aquaculture, and industrial-scale operations, representing diverse substrate characteristics and processing challenges. By including GB, BS, and NP, we aimed to address not only their importance within their respective contexts, but also their potential to contribute to sustainable practices and innovation in industry.

The research focuses on the hydrolysis process of selected fish co-products using various enzyme combinations: Alcalase 2.4L FG, Protana Prime, a sequential two-stage process, and a simultaneous combination of both enzymes. Utilizing co-products from resources that remain underexplored in hydrolysate production, this work highlights the potential of these materials. Moreover, this study aims to evaluate the degree of hydrolysis, peptide size distribution, and various biological activities of different hydrolysates, with the objective of producing protein hydrolysates with enhanced characteristics and biological properties. Combining Alcalase with Protana, an understudied exopeptidase with debittering benefits and intense umami flavor generation, this work seeks to maximize hydrolysis efficiency and provide a fresh perspective on FPH production. This approach not only offers advances in the valorization of underused marine resources but also a sustainable solution for adopting a circular economy model for FPI.

## 2. Results and Discussion

### 2.1. Hydrolysis Process and FPH Characterization

#### 2.1.1. Characterization of Co-Products and FPH–Proximate Composition

The processing of wild black scabbardfish (BS), farmed gilthead seabream (GB), and Nile perch (NP) generates significant co-products in local industries. From an average individual BS weighing 1.6 kg, the main co-products are the head and bones, representing 24.3% of the total fish weight. In the case of GB, the average weight of the fish is around 0.5 kg, with co-products primarily consisting of the carcass, making up 52.5% of it. As for NP, the co-products may have a significant expression. The Portuguese industry mainly includes trimmings, which represent approximately 1.6% of the processed fish. Approximately 1400 kg of fish is processed daily, which equates to approximately 23 kg of trimming per day.

The proximate composition of the raw materials (RM), BS heads and bones, GB carcasses, and NP trimmings, are detailed in Table 1. NP showed the highest protein content (19.3 ± 0.3%), followed by GB (16.5 ± 0.6%, *p =* 0.054) and BS (14.3 ± 1.9%, *p =* 0.0045). GB had the highest fat content, whereas the fat levels of BS and NP were similar. Moisture content was comparable in BS and NP, but significantly lower in GB (*p* = 0.0003). Ash content was highest in BS (6.9 ± 0.4%), followed by GB (5.3 ± 0.0%, %, *p =* 0.015) and NP (2.4 ± 0.16%, *p =* 0.0009). These values align with the reported values of by-products from these species; in particular, the protein content of BS was 14.9%, for GB 16.9%, and for NP 77.4% on a dry basis, which, when converted to a wet basis, are similar to the values found in the present study [25,27,28,29].

Enzymatic treatments applied to the co-products triggered significant alterations in proximate compositions, particularly in increasing protein content, resulting in FPH with average protein values of approximately 72%. The protein found in the FPH produced was consistent with those reported in the literature, which typically ranges from 60% to 91% [25,28,30]. The highest protein content was found in FPH produced from NP trimmings, which aligns with reports showing high protein content in hydrolysates from Nile perch [28,31]. Likewise, hydrolysates from gilthead seabream and black scabbardfish were also similar to values reported in literature, i.e., 73% and 76%, respectively [25,27]. Concerning the protein content variation following enzymatic treatments, it is important to note that using different combinations of enzymes (A, P, A + P, AP) resulted in notable differences in the resulting FPH from the same raw material. This finding is consistent with prior research, emphasizing the significant impact of employing various enzyme combinations during enzymatic treatments on protein content [30]. Fat content consistently decreased with enzymatic treatments, resulting in a product with reduced lipid levels, averaging 5% fat, whereas in the BS with A + P, the fat content was only 0.5%. This trend is supported by previous reports, where fat values in hydrolysates ranged from 0.2% up to 25% [28,30]. Ash content increased after enzymatic treatment due to pH adjustment during the hydrolysis process, particularly in those under alkaline conditions. Significant differences in protein, fat, moisture, and ash contents were observed among the treatments, emphasizing the biochemical diversity of the fish co-products and their influence on enzymatic hydrolysis. On this basis, it can be inferred that FPH derived from RW with a higher protein content will likely have a correspondingly higher protein concentration. This supports the conclusion that enzymatic hydrolysis is an effective method for increasing protein content and producing a lower fat product from fish co-products.

#### 2.1.2. Evolution of Degree of Hydrolysis and Yields

**Figure 1:**
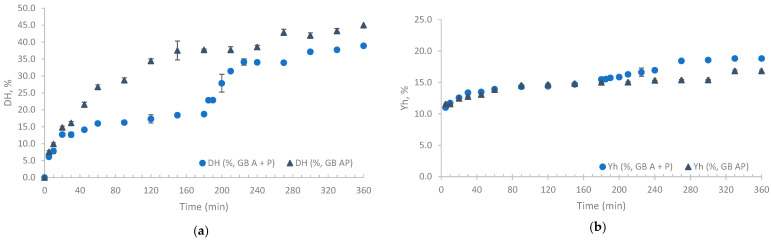
Degree of hydrolysis ((**a**), DH) and yield of the hydrolysate ((**b**), Yh) process of gilthead seabream carcass (GB) with Alcalase and Protana (AP and A + P) over time.

Alcalase 2.4L FG (A) was initially considered, because of its versatility and widespread industrial application as a producer of small bioactive peptides [32]. Combining this enzyme with others can enhance its potential, particularly when involving an exo-peptidase, as endopeptidases, such as Alcalase, create a variety of peptide lengths that can be a good substrate for exopeptidases [33]. Therefore, we introduced Protana prime (P) into the system to achieve a higher degree of hydrolysis, enhance the quantity of amino acids released, and operate at milder temperatures and pH levels [18]. An analysis of the degree of hydrolysis (DH) as a function of hydrolysis time revealed that, while the hydrolysates generated through sequential enzymatic treatment with Alcalase followed by Protana (A + P), and those obtained through combined Alcalase and Protana treatment (AP), exhibited different profiles, they consistently displayed an increasing DH, regardless of the raw materials used. This trend is exemplified in Figure 1a, which presents the hydrolysis curves for gilthead seabream carcass subjected to these enzymatic treatments.

Tailoring the enzyme selection for specific raw materials is essential. GB was observed to benefit most from the AP treatment, whereas NP and BS generally had better yields with the A + P treatment. The addition of a second enzyme resulted in a higher DH, as shown in Table 2. During the first 20 min of hydrolysis, a rapid increase in the DH was observed (Figure 1a). After this period, the DH of FPH with A + P remained stable until 180 min, while in the case of FPH prepared with AP, the DH increased considerably up to 180 min. The addition of Protana in the FPH A + P triggered a renewed boost, leading to a new phase of rapid increase that plateaued in the final phase. These observed profiles are consistent with typical enzymatic reactions carried out in two steps, as reported by García-Moreno and co-authors [30]. A similar pattern was also observed for the hydrolysates prepared with BS and NP.

At 180 min, the DH ranged from 2.4% to 41.3% (Table 2). Lower DH values were observed in hydrolysates produced using a single enzyme, particularly those using Protana. As an exopeptidase acting alone, these values were expected, since it only acts at the extremities of peptides [33]. When examining the DH of FPH prepared with AP, these consistently revealed higher values, and the maximum DH was observed with BS (41.3%). This aligns with the literature, and may indicate that hydrolysis substantially benefits from the synergistic action of both of enzymes [18]. It is worth noting that this enzyme combination has previously demonstrated the ability to achieve higher DH values, albeit not in this specific type of RM (i.e., chicken hydrolysates) [34]. However, when the reaction time was extended to 360 min, this trend was reversed, resulting in A + P hydrolysates with even higher degrees of hydrolysis, reaching values ranging from 38.9% to 57.9%. In this matter and regarding enzyme combinations, the sequential method (A + P) involves an initial stage that improves substrate accessibility for the subsequent enzyme. However, the simultaneous approach (AP) may lead to substrate competition or overlapping activity, potentially reducing the efficiency based on the protein structure of the raw material [35,36]. These observations are consistent with earlier studies highlighting the benefits of sequential enzyme systems. Such studies concluded that sequential hydrolysis utilizing both endopeptidases and exopeptidases can result in a higher DH [37,38]. This DH enhancement could potentially lower industrial production costs by maximizing the enzyme usage. Additionally, enzyme specificity is critical in peptide fractionation, as it enables the selective production of peptides of various sizes, which can be essential for customizing the product [38].

Similarly to the observations about the DH, during the first hour of hydrolysis, the yield (Yh), showed a significant increase (Figure 1b). After that, the Yh slightly increased, attaining relative stability at 180 min. In contrast, the FPH with A + P samples experienced a minor additional increase at the 180 min mark. At the end of the hydrolysis process, the Yh ranged from 3.4% to 18.8%. Notably, the two-enzyme system (A + P) with GB attained the highest yield. Regarding the protein yield (Yp), the results indicated that combined treatments (A + P and AP) typically resulted in significantly higher protein yields across all fish co-products (*p* = 0.022 and *p* = 0.047, respectively), meaning that protein conversion is higher with enzyme combinations, as expected [18]. The highest yields were observed in NP and GB with AP, with a maximum of 80% protein conversion. Single treatments (A or P) produced variable results, with A generally outperforming P. When compared to other studies in the field using similar raw materials, the yields obtained in this study fell within the expected range (20.3–57.5%), though in some treatments, they were slightly higher [8,25,27].

#### 2.1.3. Molecular Weight Distribution

The methodology used in this study focused primarily on the FPH fraction with the lowest molecular weights, excluding molecules with a weight over 10 kDa. Gel filtration chromatography profiles of hydrolysate samples were similar, and indicated the extensive hydrolysis of fish proteins, resulting mainly in small peptides (<1000 Da) and free amino acids.

When considering the distribution of molecular mass, it was evident that most of the molecular weight falls within the range of 500–100 Da representing an average of 53–54% for the FPH of BS and GB, and 74% for NP, regardless of the enzyme used. It is worth noting that, in NP hydrolysates, 7% of the compounds had a molecular weight below 100 Da, compared to 13% and 15% in BS and GB, respectively. In terms of molecular weights, the FPH can be ranked as follows: NP > BS > GB, with Nile perch hydrolysates containing the greatest percentage of high molecular weight compounds. This was expected, because higher degrees of hydrolysis (GB and BS) are associated with the FPH containing higher proportions of smaller peptides [8]. Similar results were obtained by Batista and co-authors, who reported that most peptides from enzymatically hydrolyzed black scabbardfish had molecular weights below 1000 Da [27]. In the case of GB, the available data indicate that approximately 17.6–28.5% of peptides fall below 200 Da in hydrolysates prepared with Alcalase, which aligns with the values obtained in this study [25]. Conversely, for NP hydrolysates, Wasswa and collaborators found a lower proportion (51.8%) of peptides below 500 Da [28]. These discrepancies across studies are likely due to differences in enzyme selection, reaction time, and hydrolysis conditions [8,36].

The use of different enzymatic treatments leads to unique patterns of protein fragmentation in different raw materials (Table 3).

Specifically, Alcalase tends to form smaller peptides, especially in NP, where a significant amount (68.3%) of intermediate peptides (500–100 Da) was observed. When Alcalase and Protana are used together or sequentially, a wider range of protein fragment sizes is produced, resulting in a more balanced distribution across different molecular weight ranges. These combined approaches were particularly effective in producing smaller peptides (<100 Da) in BS and GB, indicating a continuous hydrolysis process.

**Figure 2:**
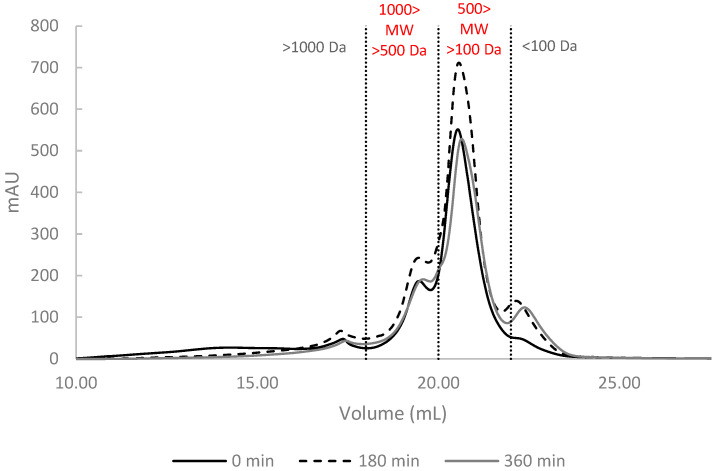
Size exclusion chromatograms of fish protein hydrolysates of gilthead seabream (GB) prepared with Alcalase and Protana (AP) at three different stages of hydrolysis (0 min, 180 min, and 360 min). Peaks separated by molecular weight ranges (>1000 Da, >500 Da, <500 Da, <100 Da).

The Figure 2 illustrates the changes in GB hydrolysates as example, highlighting the progressive evolution of molecular mass distribution over time during enzymatic treatments with AP.

Initially, larger protein fragments (>1000 Da) are predominant; however, as the hydrolysis proceeds, these high molecular weight fractions decrease, and smaller peptides become more prominent. By the middle point (180 min), there is a noticeable shift towards smaller fragments, particularly in the 500–100 Da range, as seen in NP and GB. By the end of hydrolysis, the majority of peptides fall within the smaller ranges (500–100 Da and <100 Da), indicating that prolonged hydrolysis effectively breaks down larger proteins into smaller peptides. This time-dependent breakdown is most evident in treatments with both Alcalase and Protana, where sequential hydrolysis results in a more even distribution of small and intermediate peptides by the end of the reaction.

### 2.2. Antioxidant Activity

The antioxidant activities of all FPH were evaluated through 2,2-diphenyl-1-picrylhydrazyl (DPPH) and 2,2′-azino-bis(3-ethylbenzothiazoline-6-sulfonic acid (ABTS) radical scavenging assays by calculating the half-maximal effective concentration (EC_50_). Additionally, the reducing power was assessed by comparing the concentrations needed to achieve an absorbance of 0.5 (Abs = 0.5).

**Figure 3:**
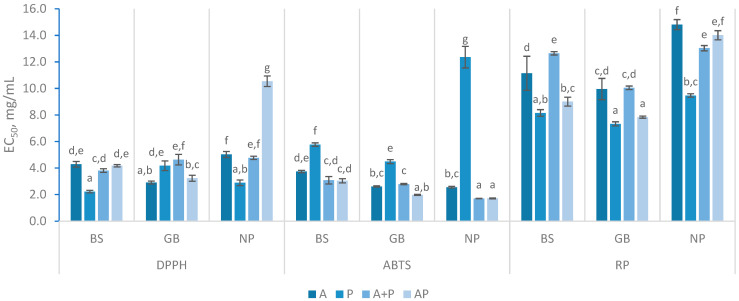
Antioxidant assays (DPPH, ABTS, reducing power (RP)) with different hydrolysates prepared with black scabbardfish heads and bones (BS), gilthead seabream carcass (GB) and Nile perch trimmings (NP) using various enzyme combinations: Alcalase (A), Protana (P), Alcalase followed by Protana (A + P), and Alcalase and Protana added simultaneously (AP). The columns represent the mean values, and different letters represent significant differences (*p* < 0.05).

For DPPH scavenging, enzymatic treatments exhibited varying levels of effectiveness within the FPH produced (Figure 3), revealing no clear pattern, other them a concentration-dependent behavior, which was previously observed by Wang and coworkers [21]. The best result was obtained with BS using Protana (P), with an EC_50_ of 2.23 ± 0.09 mg/mL. Similar values were observed in GB treated with Alcalase and NP with Protana. Regarding enzyme combinations, A + P yielded the best results in BS and NP, with the scavenging capacity improving between 180 min and 360 min. This supports the idea that prolonged enzymatic treatment can enhance radical scavenging efficiency, as more peptide bonds are cleaved, leading to more efficient radical scavenging over time [27]. Furthermore, the addition of another enzyme in the system in a second stage may lead to an increase in radical scavenging capacity, as reported in the literature [30]. The simultaneous use of Alcalase and Protana (AP) demonstrated less efficient DPPH scavenging, though improvements were observed with longer reaction times. FPH from GB with AP had the lowest EC_50_ value at 180 min (3.68 ± 0.2 mg/mL), which further decreased to 3.24 ± 0.23 mg/mL at 360 min. Wang and colleagues have demonstrated that the size of antioxidant peptides is a critical factor influencing their activity [21]. As hydrolysis time increases, a corresponding increase in DH occurs, resulting in the production of smaller peptide fractions. However, despite these enhancements, the results remained different than previously reported findings for FPH of gilthead seabream, where the scavenging activity reached around 50% at a concentration of 1.44 mg/mL [25]. Among the raw materials tested, BS and GB exhibited the most effective DPPH scavenging activity, with generally lower EC_50_ values than NP hydrolysates. Although the results for all FPH varied substantially, they fell within the expected range of DPPH values reported in the literature. Several authors have documented EC_50_ values ranging from 1 to 10 mg/mL, with some studies even reporting values below 0.3 mg/mL [8,39,40,41]. Such differences are mainly attributed to variations in hydrolysis conditions and refining processes; thus, significant variability in the outcomes is expected, given the diverse treatments methods used [30].

The ABTS scavenging activity in BS samples with AP treatment had the lowest EC_50_ at 180 min (2.85 ± 0.19 mg/mL), which slightly increased to 3.04 ± 0.16 mg/mL at 360 min. The A + P treatment showed similar trends but in slightly higher concentrations, as seen in the Figure 3. The opposite was observed in the GB and NP samples, where the ABTS scavenging activity was higher at 360 min (lower concentrations of EC_50_). A + P and AP treatments had similar values of EC_50_ within the same raw materials, except in GB, where a distinction was observed between A + P and AP, with the latter being higher. The ABTS scavenging capacity was more consistent for all FPH samples, with EC_50_ values ranging from 1.71 to 3.74 mg/mL. The values achieved in this study were consistent with those reported for hydrolysates of by-products from Cape hake and salmon using Alcalase, which ranged from 2.1 to 2.4 mg/mL. Additionally, they align with results from various FPH, where concentrations ranged between 1.12 and 4.93 mg/mL [8,40]. Furthermore, FPH treated only with Protana consistently underperformed, with EC_50_ values ranging from 4.49 ± 0.10 mg/mL to 12.36 ± 0.81 mg/mL, suggesting that Protana may not be as effective in enhancing ABTS scavenging compared to other enzyme treatments.

The DPPH radical is stable in lipophilic media, while the ABTS^•+^ is stable in water-based solutions, but both are used to measure the presence of hydrogen atoms or electron-donating substances [42,43]. During hydrolysis, smaller peptides and individual amino acids were produced based on the specificity of enzymes involved, as previously discussed. These were consistent with previous research, showing that antioxidant capacity, particularly regarding ABTS and DPPH scavenging activities, were strongly influenced by the molecular size and amino acid composition of peptides, with lower molecular weight peptides exhibiting greater radical scavenging abilities [21,44,45]. Also, the results showed that Protana hydrolysates were more effective in capturing the DPPH^•^, suggesting the presence of less water-soluble peptides, formed by exposure of hydrophobic regions in the proteins [46]. However, when Alcalase was introduced into the system, an overall improvement in the ability to capture the ABTS^•+^ was observed, indicating a possible formation of more hydrophilic peptides with this enzyme.

The reducing power assay typically involves the reduction of ferric ions (Fe^3^⁺) to ferrous ions (Fe^2^⁺) [47]. This assay (Figure 3) revealed that the GB hydrolysate presented a higher RP capacity, requiring an average of 9.81 mg/mL to reach 0.5 in absorbance, compared to 10.98 mg/mL for BS and 12.69 mg/mL for NP. The results obtained were within the same range as the findings reported in previous research [8,48]. For instance, an A_0.5_ value of 15.0 mg/mL was obtained for Cape hake protein hydrolysates, while A_0.5_ values ranging from 10.0 to 31.25 mg/mL were observed in FPH prepared from various discarded species and by-products [30,49]. In contrast, lower values, ranging from 3.19 to 6.35 mg/mL, have been documented for different hydrolysates derived from fish and fish parts [40]. In this study, for BS, the FPH with A + P treatment had the lowest reducing power at 180 min (12.73 ± 0.41 mg/mL), with minimal change over time. However, the AP treatment, although similar in the initial phase, showed a higher reducing capacity which switched from 12.20 ± 0.09 mg/mL to 9.01 ± 0.34 mg/mL. GB hydrolysates treated with AP for over 360 min exhibited stronger reducing power (7.84 ± 0.08 mg/mL) compared to those treated with P. The other GB hydrolysates exhibited similar values of approximately 10 mg/mL. As for NP, the reducing power decreased significantly with both A + P and AP treatments over time (*p =* 0.028 and *p =* 0.005 respectively), showing a different behavior than its analog’s FPH. Similar to the DPPH activity results, FPH prepared with Protana showed the best results in the reducing power assay. The results also indicated that the simultaneous application of Alcalase and Protana (AP) generally enhanced the antioxidant properties of FPH. Among the raw materials, GB consistently exhibited the highest antioxidant activity. Regarding the influence of reaction time, the results were inconclusive, contrasting with some studies that concluded that antioxidant activity increases with a high degree of hydrolysis [27,30].

### 2.3. Metal Chelating Activities

**Figure 4:**
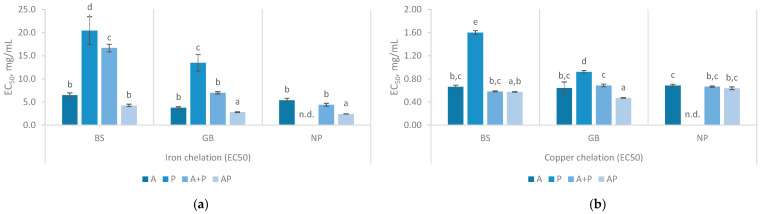
Iron (**a**) and cooper (**b**) chelation activity with different hydrolysates prepared with black scabbardfish heads and bones (BS), gilthead seabream carcass (GB) and Nile perch trimmings (NP) using various enzyme combinations: Alcalase (A), Protana (P), Alcalase followed by Protana (A + P), and Alcalase and Protana added simultaneously (AP). n.d. indicates values that could not be determined for the samples. The columns represent the mean values, and different letters reveal significant differences (*p* < 0.05).

The capability to interact with transition metals such as copper and iron is also an important indicator of antioxidant activity. Transition metal ions, such as Fe^2^⁺ and Cu^2^⁺, catalyze the formation of reactive oxygen species, like the hydroxyl radical, which induce lipid peroxidation [30,50,51,52]. On the other hand, the chelation of these metals is extremely important to promote their absorption [53]. In the iron chelation assays, the EC_50_ values ranged widely, as shown in the Figure 4a. For example, the EC_50_ value was 2.40 ± 0.04 mg/mL when using NP and AP, while it was nearly ten times higher when BS and P was used. The Protana hydrolysates performed poorly in this analysis, with all the raw materials showing lower effectiveness or producing values that did not allow the calculation of EC_50_.

The study found that, in general, hydrolysates from BS had the lowest iron-chelating activity. When Alcalase and Protana (AP) were used, the EC_50_ decreased from 5.59 ± 0.26 mg/mL to 4.24 ± 0.26 mg/mL over 360 min, indicating enhanced chelation capacity as the reaction progressed. However, the A + P treatment resulted in a significant decrease in chelation efficiency (16.66 ± 0.83 at 360 min). GB and NP hydrolysates showed the highest iron chelation activity, with AP yielding the lowest EC_50_ values (2.79 ± 0.07 and 2.40 ± 0.08 respectively). The results obtained were comparable to those reported by Naghdi and co-authors, which found that FPH from tuna processing by-products exhibits iron-chelating activity that increases with dosage, achieving 30–55% chelation at 4 mg/mL [52]. AP hydrolysates demonstrated high effective chelation, but their EC_50_ values were considerably higher from those reported in studies by Pires and collaborators and Henriques and co-authors, where EC_50_ values ranged from 0.26 to 0.53 mg/mL [8,40].

Concerning the copper chelation activities (Figure 4b), Protana hydrolysates had a behavior similar to that observed in the chelating activity of iron. In the remaining hydrolysates (i.e., A, A + P, and AP), EC_50_ concentrations are very similar, always presenting values below 1 mg/mL. However, the GB hydrolysate produced with AP achieved the lowest EC_50_ value (0.47 ± 0.01 mg/mL). These values were considerably lower than those presented in the literature, where a range between 2.49 and 5.66 mg/mL in FPH were obtained from different by-products [40]. Nevertheless, some studies on FPH from salmon heads and Cape hake by-products mention similar results around 0.64 mg/mL [8].

In the chelating activities, the results indicate that the sequential application of Alcalase and Protana (AP) tends to enhance both iron and copper chelation activities. Notably, the hydrolysates studied were far more effective in copper chelation activity than in iron chelation activity. Some researchers have proposed that the rise in metal-chelating activity is related with the increased DH. Low molecular weight peptides have stronger charges, particularly in carboxyl groups, and higher mass-to-charge ratios, enabling them to participate more effectively in metal complexation [52].

### 2.4. Biological Activities

**Figure 5:**
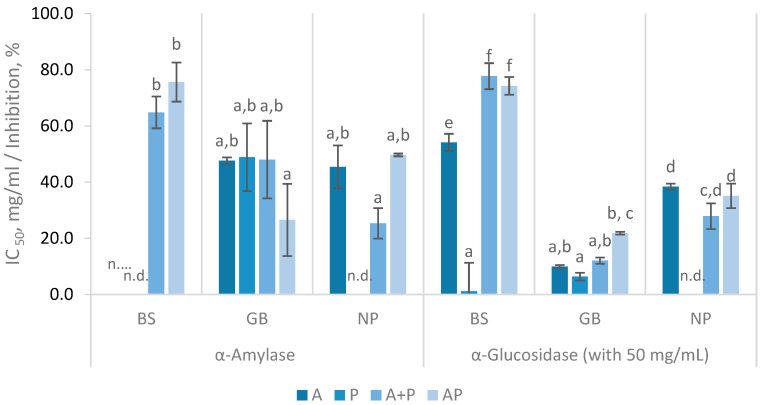
IC_50_ values for α-Amylase inhibitory activity and α-glucosidase inhibition percentages (%) with 50 mg/mL of FPH prepared from black scabbardfish heads and bones (BS), gilthead seabream carcass (GB) and Nile perch trimmings (NP) using various enzyme combinations: Alcalase (A), Protana (P), Alcalase followed by Protana (A + P), and Alcalase and Protana added simultaneously (AP). ‘n.d.’ indicates values that could not be determined for the samples. The columns represent the mean values, and different letters indicate significant differences (*p* < 0.05).

Fish protein hydrolysates were tested for their biological activities to determine their potential as antidiabetic, anti-Alzheimer’, and antihypertensive agents. These activities were established based on their ability to inhibit α-amylase and α-glucosidase for antidiabetic activity, acetylcholinesterase (AChE) for anti-Alzheimer activity, and angiotensin-converting enzyme (ACE) for antihypertensive activity. AChE and α-glucosidase inhibition percentages were measured at concentrations of 50 mg/mL, ACE inhibition was tested at 5 mg/mL and α-amylase activity was determined using the half-maximal inhibitory concentration (IC_50_). The results (Figure 5) highlight the differences between the enzymatic treatments and raw materials.

Both α-amylase and α-glucosidase are essential enzymes for glucose metabolism. α-Amylase aids in digestion by breaking down dietary starch into oligosaccharides, whereas α-glucosidase breaks down disaccharides into glucose for absorption [46]. Thus, the inhibition of these enzymes is an effective strategy for managing glucose levels in organisms. In the α-amylase inhibitory assay (Figure 5), IC_50_ values were only determined for some samples, as BS (A), BS (P) and NP (P) FPH did not reach the IC_50_, plateauing at lower inhibition percentages. Nevertheless, FPH showed a concentration-dependent inhibitory effect, consistent with previous studies [8]. GB hydrolysates treated with Alcalase and Protana (AP) exhibited an IC_50_ of 23.86 ± 3.52 mg/mL at 180 min, which increased to 26.58 ± 12.83 mg/mL at 360 min. This treatment produced higher α-amylase inhibitory activity, along with NP hydrolyzed with A + P, although the differences were not statistically significant (*p* > 0.05). For NP hydrolysates, α-amylase inhibition was observed, with a notable result of 25.36 ± 5.4 mg/mL in the A + P treated samples at 360 min. The inhibition of α-amylase using 20 mg/mL of FPH prepared with various enzymes (Alcalase, papain, and pepsin) has been reported to range from 16.61% to 45.71% [46]. These results were similar to those obtained in this study, where 50% inhibition was observed at concentrations of 23–26 mg/mL. Other studies have reported IC_50_ values spanning a broad range from 5.70 to 84.37 mg/mL [40].

The α-glucosidase inhibition (Figure 5) varied completely among the different fish co-products, with BS hydrolysates presenting the highest α-glucosidase inhibition and GB the lowest. In the case of BS, the use of A + P and AP resulted in an enhanced inhibition of 77.76 ± 4.62% and 74.3% ± 3.17%, respectively. For GB, all treatments showed limited α-glucosidase inhibition, yet AP treatment provided the highest inhibition (21.83% ± 0.51%). As for NP, the highest α-glucosidase inhibition was observed in the FPH prepared with A (38.42 ± 1.13%) and AP (35.14 ± 4.35%) treatments. FPH has been reported to have a weak inhibitory effect on α-glucosidase [46]. Values above 100 mg/mL in IC_50_ were expected according to the results reported by Henriques and co-authors for FPH produced with Alcalase from several discarded fish and by-products [40]. Amini Sarteshnizi and collaborators referred a maximum 16.66% inhibition at 20 mg/mL of FPH from Sardinella produced with Alcalase [46].

**Figure 6:**
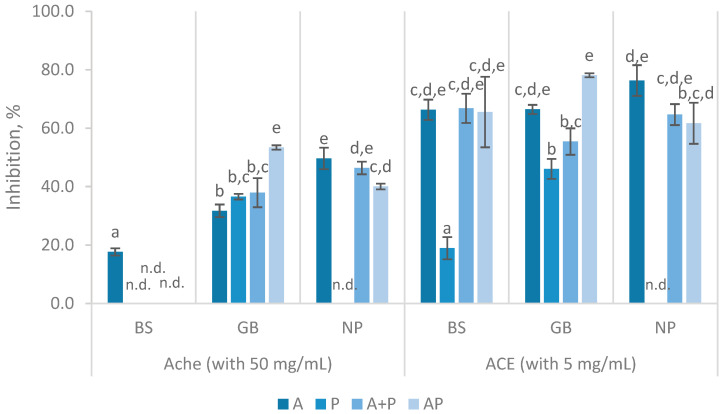
AChE inhibition percentages (%) with 50 mg/mL and ACE inhibition percentages (%) with 5 mg/mL of FPH prepared from black scabbardfish heads and bones (BS), gilthead seabream carcass (GB) and Nile perch trimmings (NP) using various enzyme combinations: Alcalase (A), Protana (P), Alcalase followed by Protana (A + P), and Alcalase and Protana added simultaneously (AP). ‘n.d.’ indicates values that could not be determined for the samples. The columns represent the mean values, and different letters significant differences (*p* < 0.05).

Similar to the observed in α-glucosidase inhibitory activity, AChE inhibitory activity was also limited in the produced FPH, as shown in Figure 6. The highest inhibition was observed in the GB hydrolysate when treated with the simultaneous AP, reaching over 50% inhibition, followed closely by a similar result in NP with A. Concerning the BS FPH, only the hydrolysates prepared with Alcalase inhibited AChE, reaching 17.69 ± 1.26% with 50 mg/mL. GB hydrolysates demonstrated inhibition levels of 31.73 to 54.15%, whereas NP hydrolysates showed inhibition ranging from 40.04 to 49.65%. AChE inhibition levels observed in this study align with previous findings, although variations in inhibition can be observed depending on the hydrolysis conditions. Indeed, AChE activity inhibition has been reported to range from 18.11 to 40.45% in FPH prepared from tilapia with Alcalase at concentration of 50 mg/mL [54]. Conversely, for anchovy hydrolysates prepared with Alcalase, papain, and pancreatin, FPH concentrations ranged from 100 to 400 mg/mL, resulting in inhibition percentages between 11.78% and 60.65% [55]. Similar to most studies on the AChE inhibitory activity of FPH, none of the hydrolysates analyzed in this study exhibited particularly strong inhibition potential against AChE [54,55].

ACE inhibition had similar values in all FPH, with inhibition levels above 50%, except for those prepared using Protana (Figure 6). The results with BS indicated that neither the A + P nor AP treatments led to an increase in activity compared to the enzymatic treatment with Alcalase alone. GB hydrolysates with AP had the highest ACE inhibition (78.12 ± 0.69%); however, this value was not significantly (*p =* 0.285) different from that obtained in FPH with A (66.42 ± 4.65%). For NP, A was highly effective (76.32 ± 5.25%), but its inhibition decreased with the addition of Protana (A + P), resulting in 64.65 ± 3.58% inhibition. The ACE inhibition results from the current study’s alignment with prior research, although with slight differences in inhibition levels that can be attributed to variations in protein sources [40]. Pires and co-authors reported an IC_50_ of 0.86 to 2.2 mg/mL for Alcalase hydrolysates that exhibited ACE inhibitory activity in a concentration-dependent manner, directly comparable to the three FPH prepared with A in the present study, which displayed an inhibition between 66.30 and 76.32% [8]. Furthermore, other FPH obtained from various discarded fishes and by-products showed similar ACE inhibitory activity (61.20–85.95%), at 5 mg/mL, to that reported in this study [40]. ACE inhibitory activity in freshwater carp hydrolysates showed inhibition ranging from 43% to 71% at 5 mg/mL, depending on the enzyme specificity and degree of hydrolysis [56]. In a different study involving tuna by-products, Suo and colleagues reported IC_50_ values of 0.48, 0.59, and 0.76 mg/mL. The low IC_50_ concentrations observed were attributed to the strong binding affinity of ACE for small and specific peptides, facilitated by hydrogen bonds, electrostatic forces, and hydrophobic interactions, suggesting a mechanism that may explain the overall levels of inhibition noted [57]. As the FPH obtained in this study was primarily composed of low molecular weight peptides, a similar phenomenon was expected. A study reported that hydrolysates produced from monkfish using single enzymes (Alcalase or Neutrase) and a double-enzyme system (Alcalase followed by Neutrase) showed that the hydrolysate prepared with the double-enzyme system exhibited higher ACE inhibitory activity (53.22 ± 2.63% at 2.5 mg/mL) compared to those produced with single enzymes. This suggests that using a combination of enzymes enhances the release of bioactive peptides with improved ACE inhibitory properties, as observed in this study [58]. These authors suggested that higher enzyme specificity could contribute to higher inhibition, highlighting how enzyme type, substrate, and hydrolysis conditions create peptides with varying molecular weights and amino acid profiles, which significantly influence ACE inhibition [40,56,57,58].

## 3. Materials and Methods

### 3.1. Raw Materials

Frozen co-products that were generated during the portioning of fish within frozen fish processing industries served as the raw material for this study. Artesanal Pesca Lda. (Sesimbra, Portugal), provided the heads and bones of wild black scabbardfish (*Aphanopus carbo*) after filleting. Vivid Food, Lda. (Vila Nova da Barquinha, Portugal), supplied the carcass of gilthead seabream (*Sparus aurata*) from aquaculture obtained after filleting. The trimming of Nile perch (*Lates niloticus*) was obtained from GelPeixe Lda. (Lisboa, Portugal). The co-products were received frozen, and after thawing, the material was crushed and homogenized using an industrial meat grinder (HOBART, Troy, OH, USA) and knife mill grinder (Grindomix GM200, Retch GmbH, Haan, Germany). Next, the material was vacuum packed and stored at −20 °C until further utilization.

### 3.2. Chemicals and Reagents

Food-grade Alcalase 2.4L^®^ and Protana Prime^®^ were generously provided by Novozymes (Bagsvaerd, Denmark). o-Phthaldialdehyde (OPA), 3-(2-pyridyl)-5,6-bis(4-phenyl-sulfonic acid)-1,2,4-triazine (ferrozine), 2,2-azinobis-(3-ethylbenzothiazoline-6-sulfonic acid (ABTS), 2,2-diphenyl-2-picrylhydrazyl (DPPH), cytochrome c from bovine heart, hexaglycine, triglycine, glycine, α-Amylase from porcine pancreas (EC 3.2.1.1), α-Glucosidase from Saccharomyces cerevisiae (EC 3.2.1.20), Acetylcholinesterase from electric eel (EC 3.1.1.7), Angiotensin-converting enzyme (ACE) from rabbit lung (EC 3.4.15.1), Acarbose, Berberine, Captopril, Hippuryl-l-histidyl-leucine (HHL), and Hippuric acid (HA) were purchased from Sigma-Aldrich (St. Louis, MO, USA). Ethylenediaminetetra-acetic acid (EDTA) standard certified for nitrogen calibration was purchased from the LECO Corporation (St. Joseph, MI, USA). Ribonuclease A from bovine pancreas and aprotinin from bovine lung were purchased from Cytiva (Washington, DC, USA). All other chemicals used were of analytical grade.

### 3.3. Proximate Composition

The ash and moisture content of the different raw materials and fish protein hydrolysates (FPH) were analyzed according to AOAC standard methods [59]. The protein content was determined using an FP-528 LECO nitrogen analyzer (LECO, St. Joseph, MI, USA) calibrated with EDTA (nitrogen = 9.57 ± 0.03%) according to the Dumas method [60]. The lipid content was measured using the Folch methodology described by Sapatinha and co-authors [61].

### 3.4. Enzymatic Hydrolysis

The enzymatic hydrolysis experiment was conducted at lab-scale using two proteolytic enzymes: Alcalase, an endoprotease; and Protana, an exoprotease. Both enzymes were tested individually as well as in combination, as represented in Figure S1, using simultaneous and two-stage addition methods. The reaction was carried out in a 5 Liter glass reactor with a controlled pH and agitation at 300 rpm, using a 1:2 ratio of raw material to distilled water. The pH, time, temperature, and enzyme ratio used in the reaction are displayed in Table 4. The enzymatic settings were selected based on the optimum operating conditions recommended by Novozymes for maximum enzyme activity [18,62].

Aliquots of 100 mL were collected at different intervals during hydrolysis (5, 10, 20, 30, 45, 60, 90, 120, 150, 180, 185, 190, 200, 210, 225, 240, 270, 300, 330, 360 min). For hydrolysis performed with the two enzymes (AP), after the first hour of hydrolysis, samples were collected every 30 min. The hydrolysates were inactivated at 90 °C for 10 min, cooled and centrifuged (10,000× *g*, 20 min, 4 °C). Next, the supernatant composed by free fat and aqueous hydrolysate was decanted. The aqueous fraction was freeze-dried and pulverized to obtain the FPH. The samples were stored at −20 °C until further analyses.

### 3.5. Fish Protein Hydrolysates Characterization

#### 3.5.1. Yields

The hydrolysis and protein yields of different hydrolysis processes were calculated using the following equations [8]:

(1) $$Hydrolysis\;yield\;\left(\%\right)=\frac{W_{f}}{W_{i}}\times 100$$

(2) $$Protein\;yield\;\left(\%\right)=\frac{P_{f}}{P_{i}}\times 100$$

In these equations, W_f_ represents the weight in grams (dw) of the FPH, and W_i_ is the weight in grams (dw) of the raw material. Similarly, P_f_ is the total protein content (in grams) of FPH, and P_i_ is the total protein content (in grams) in the raw material.

#### 3.5.2. Degree of Hydrolysis

To determine DH, the o-phthalaldehyde (OPA) method, outlined by Nielsen and co-authors, was employed [63]. The assay was carried out at room temperature. Four hundred microliters of samples (0.5 mg/mL) were added to 3 mL of OPA solution. The mixture was incubated for 2 min, and the absorbance was read at 340 nm using an Evolution 201 UV–Visible Spectrophotometer (Thermo Scientific, Waltham, MA, USA). A blank was prepared using distilled water. A control with serine 0.1 mg/mL was also prepared, likewise. The degree of hydrolysis was calculated using the following formula:

(3) $$DH\left(\%\right)=\left[\frac{A_{sample}-A_{blank}}{A_{serine}-A_{blank}}\times \frac{\left(0.9516\times 10\right)}{W\times N\times 6.25}-\beta \right]\times \frac{100}{8.6}$$

where A_sample_ is the absorbance of FPH, A_blank_ is the absorbance of the blank, A_serine_ is the absorbance of the serine solution, W is the weight in grams of hydrolysate, N is the total nitrogen content (%) of FPH, and β is a constant (0.4), determined to fish as raw material, according to Nielsen et al., 2001 [63].

#### 3.5.3. Molecular Weight Distribution (MWD)

The molecular mass distribution of the different FPH was determined using gel filtration chromatography on a FPLC ÄKTA system (Amersham Biosciences, Uppsala, Sweden) with a Superdex Peptide 10/300 GL column and a UV detector at 254 nm. The samples were filtered (0.22 µm) and loaded into a 100 µL loop. The eluent consisted of 30% acetonitrile with 0.1% trifluoroacetic acid at a flow rate of 0.5 mL/min. A molecular mass calibration curve was generated using the following standards: ribonuclease A (13,700 Da), cytochrome C (12,384 Da), aprotinin (6500 Da), angiotensin I (1296 Da), hexaglycine (360 Da), triglycine (189 Da), and glycine (78 Da). The molecular weight distribution of the peptides was estimated by dividing the area of each identified peak by the total area of all peaks [8].

### 3.6. Determination of Antioxidant Activity

#### 3.6.1. DPPH Radical Scavenging Activity

To determine antioxidant activity, FPH samples were prepared at concentrations ranging from 0.5 to 20 mg/mL in water. To determine DPPH radical scavenging, the method of Shimada and co-authors was employed, with adjustments and using an Evolution 201 UV–Visible Spectrophotometer (Thermo Scientific, Waltham, MA, USA). A control sample was prepared, using Milli-Q water instead of the FPH solution [8,42,64].

#### 3.6.2. ABTS Radical Cation Scavenging Activity

ABTS radical scavenging activity was evaluated according to Re and collaborators, using an ABTS radical cation solution, and a control sample was prepared using distilled water instead of the sample [8,65].

#### 3.6.3. Reducing Power (RP)

The RP of FPH was measured by the method described by Oyaizu, with the modifications indicated by Pires and co-authors [8,47]. A control was prepared with distilled water instead of the sample. The concentration, corresponding to an absorbance value of 0.5 (A_0.5_), was calculated for each hydrolysate.

### 3.7. Metal Chelating Activities

#### 3.7.1. Cu^2+^-Chelating Activity

To measure the copper-chelating activity, the methodology described by Torres-Fuentes and co-authors, with slight modifications, was used [8,51]. A control was prepared similarly, using distilled water instead of the sample solution.

#### 3.7.2. Fe^2+^-Chelation Activity

The determination of iron-chelating activity was tested at concentrations ranging from 0.5 to 20 mg/mL. The methodology used was undertaken according to the method described by Decker and Welch, with the modifications presented in Pires et al., and using a control prepared with distilled water instead of the sample solution [8,50].

The percentage inhibition of each antioxidant and chelating activities, as previously described, was calculated according to the following equation:

(4) $$Inhibitory\;activity\;(\%)=\frac{A_{cantrol}-A_{Sample}}{{Abs}_{cantrol}}\times 100$$

where A_sample_ and A_control_ correspond to the absorbance of the sample and the control, respectively. The EC_50_ value was determined for each hydrolysate, and all measurements were performed in triplicate. The results are presented as mean values ± standard deviation.

### 3.8. Determination of Biological Activity

#### 3.8.1. α-Amylase Inhibitory Activity

For α-amylase inhibitory activity, a concentration-dependent effect was tested for all FPH. The samples were prepared at concentrations ranging from 10 to 100 mg/mL in 0.1% dimethyl sulfoxide (DMSO). The enzyme substrate, starch-dyed with Remazol Brilliant Blue R, was boiled in 1% of Tris-HCl buffer for 5 min, and then cooled. The assay was initiated by adding 200 µL of the sample to 100 µL of porcine pancreatic α-amylase (PPA), prepared at 0.1 U/mL in 0.5 M Tris-HCl buffer, with 0.01 M CaCl₂ and pH 6.9 (A). After pre-incubation at 37 °C for 20 min with shaking, 100 µL of the dyed starch substrate was added, followed by incubation (37 °C for 10 min with shaking). The reaction was stopped by adding 500 µL of 50% acetic acid. The mixture was then centrifuged (5000 rpm, 5 min), and the absorbance of the supernatant was measured at 595 nm using an Evolution 201 UV–Visible Spectrophotometer. Simultaneously, a negative control (0−) was prepared by replacing the sample with 0.1% DMSO, and the enzyme with a buffer. A positive control (0+) was obtained by substituting the sample with 0.1% DMSO. A blank (B) was prepared for each sample by changing the enzyme with the buffer. All the other non-mentioned steps were unchanged [8,66].

#### 3.8.2. α-Glucosidase Inhibitory Activity

The α-glucosidase inhibitory activity was assessed by testing the FPH, prepared at 50 mg/mL in a potassium phosphate buffer (0.1 M, pH 6.9). The FPH solution was pre-incubated with 100 µL α-glucosidase (0.25 U/mL) at 37 °C for 30 min. Following pre-incubation, 50 µL of p-NPG (5 mM, p-nitrophenyl–α-D-glucopyranoside in buffer) was added. The samples were incubated at 37 °C for 5 min, and the absorbance was measured at 415 nm using a microplate reader (Bio Rad Model 680, Hercules, CA, USA). Similar to the previous inhibitory activity, a negative control (0−) was prepared by replacing both the sample and enzyme with the buffer. A positive control (0+) was also prepared by replacing the sample with the buffer. A blank was also prepared for each sample by exchanging the substrate with the buffer. All other steps remain unchanged, unless specified otherwise. Higher concentrations could not be tested because of the solubility limitations of the hydrolysates in this assay, which prevented the preparation of higher concentrations needed to measure the IC_50_ for this activity [67].

For both the α-glucosidase and α-amylase inhibitory activity assays, acarbose was used as a standard commercial inhibitor, at a concentration of 10 mg/mL. The assays were performed in quadruplicate and the results are presented as mean values ± standard deviation. The percentage inhibition was calculated as follows:

(5) $$Inhibitory\;activity\;(\%)=\frac{\left(C^{+}-C^{-}\right)-\left(A-B\right)}{\left(C^{+}-C^{-}\right)}\times 100$$

where A is the absorbance of the test sample (assay with hydrolysate and enzyme), B is the absorbance of the sample blank, C^+^ is the absorbance of the positive control, and C^−^ is the absorbance of the negative control blank.

#### 3.8.3. Acetylcholinesterase (AChE) Inhibitory Activity

The FPH samples were prepared at a concentration of 50 mg/mL in water for the assessment of Acetylcholinesterase (AChE) inhibitory activity. The assay was performed with AChE from electric eel, at a concentration of 0.36 U/mL. Briefly, 40 µL of enzyme was preincubated (37 °C, 15 min) with 80 µL of sodium phosphate buffer (100 mM with 1 mM EDTA, pH 8.0) and 40 µL of the test sample. The control reaction was performed by changing the volume of the sample with Milli-Q water. Following pre-incubation, 40 µL of a substrate solution containing 0.3 mM acetylthiocholine and 0.25 mM DTNB (and 5,5′-dithio-bis-(2-nitrobenzoic acid) (DTNB) in buffer was added to each well. The reaction was monitored by measuring the absorbance at 415 nm for 5 min with readings taken at 20 s intervals [68]. As a standard commercial inhibitor, 100 µM berberine prepared in 10% ethanol was used and subjected to the assay following the same procedure as the sample. The assay was performed in quadruplicate, and the results are presented as mean values ± standard deviation. The percentage inhibition was calculated using the following formula:

(6) $$AChE\;Inhibitory\;activity\;(\%)=1-\frac{V_{Sample}}{V_{Control}}\times 100$$

where V_sample_ is the reaction rate in the presence of the test sample, and V_control_ is the reaction rate of the control.

#### 3.8.4. Angiotensin-Converting Enzyme (ACE) Inhibitory Activity

The ACE inhibitory activity using HHL as substrate was evaluated by high performance liquid chromatography (HPLC) [8,69]. Briefly, 10 µL of FPH solution (5 mg/mL), were mixed with 10 µL of 0.2 U/mL ACE prepared in a sodium borate buffer (100 mM with 300 mM NaCl, pH 8.3). The mixture was pre-incubated at 37 °C for 20 min, after which 50 µL of HHL (2.17 mM in buffer) were added, and the mixture was incubated at 37 °C for 30 min. A control reaction was made by changing the volume of sample with sodium borate buffer. The reaction was stopped by adding 85 µL of 1 M HCl, and the solution was filtered. A 10 µL aliquot was injected into an HPLC (Agilent 1260 Infinity II; Agilent Technologies, Santa Clara, CA, USA) equipped with a reversed-phase C18 column (4.6 × 100 mm, 2.7 μm, InfinityLab Poroshell 120 EC-C18). The identity of the HA and HHL was assessed by comparison with the retention times of the standards. Peak areas were obtained using ChemStation software (LTS 01.11) for LC (Agilent Technologies, Santa Clara, CA, USA). The assay was performed in triplicate, and the results are presented as mean values ± standard deviation. The percentage of ACE inhibition was calculated as follows:

(7) $$\mathrm{ACE}\;\mathrm{inhibition}\;(\%)=\frac{{HA}_{control}-{HA}_{Sample}}{{HA}_{control}}\times 100$$

where HA_Control_ is the concentration of HA in the reaction with the buffer instead of the sample, and HA_sample_ is the concentration of HA in the reaction with the sample. Captopril (21.7 µg/mL) concentration was used as the commercial inhibitor.

### 3.9. Statistical Analysis

The results of the analysis are presented as mean values ± standard deviation (SD). All statistical analyses were conducted using STATISTICA© software version 12, developed by StatSoft, Inc. (Tulsa, OK, USA). The data were assessed for normality of distribution and homogeneity of variances using the Shapiro–Wilk and Levene’s tests, respectively. Differences between the mean values of the groups were analyzed using a one-way analysis of variance (ANOVA), followed by a Tukey’s HSD multiple comparison test. A *p*-value < 0.05 was considered statistically significant.

## 4. Conclusions

The study successfully produced FPH from frozen fish industry co-products, demonstrating their potential as valuable sources of bioactive compounds, and offering a feasible approach to apply in fish processing for “future by-products”. Employing enzymatic hydrolysis on co-products from black scabbardfish, gilthead seabream, and Nile perch demonstrates promising biological activities, particularly antioxidant and antihypertensive properties. The use of Alcalase and Protana in both single-stage and sequential methods effectively maximized hydrolysis yields and enhanced bioactive potential, even with hydrolysis limited to 180 min. The hydrolysates exhibited high degrees of hydrolysis, significant protein yields, and a lower molecular weight distribution, correlating with an enhanced biological activity. These properties make the hydrolysates attractive for numerous applications. The biological activities observed—especially the antioxidant and antihypertensive—underscore the health-promoting potential of these FPH. While the antihypertensive properties indicated effective ACE inhibition at low concentrations, antioxidant assays further validated the capacity of FPH to counteract oxidative stress.

This work offers a blueprint for utilizing and valorizing fish processing co-products, which are typically low in value and costly to dispose of, even though they may become a source of functional ingredients with significant market potential in the food, agriculture, and nutraceutical sectors. The enzymatic approaches explored align with sustainable development goals by reducing waste, maximizing resource efficiency, improving nutrition, and promoting sustainable fish industry and agriculture. The further identification of specific peptides and improvements in sensory attributes such as color, odor, and taste will support their broader acceptance and applicability in industrial formulations, advancing circular economy models and sustainable waste management solutions.

## Figures and Tables

**Table 1:** Proximate composition of black scabbardfish heads and bones (BS), gilthead seabream carcass (GB) and Nile perch trimmings (NP) co-products, raw materials (RM, ww), and their final hydrolysates (dw) produced with different enzyme combinations (A, P, A + P and AP), in terms of protein, fat, moisture and ash content. Values correspond to average ± SD for n = 3. Different upper cases in the RM samples indicate statistical differences (*p* < 0.05). Different lower cases in the remaining samples indicate statistical differences between hydrolysates (*p* < 0.05).

Co-Products		Protein, %	Fat, %	Moisture, %	Ash, %
BS	RM	14.3 ± 1.9 ^A^	8.1 ± 0.3 ^A^	71.0 ± 0.1 ^B^	6.9 ± 0.4 ^C^
	A	77.1 ± 0.1 ^g^	2.3 ± 0.1 ^a,b^	5.4 ± 0.1 ^b^	15.2 ± 0.0 ^d^
	P	56.8 ± 0.2 ^a^	n.d.	n.d.	n.d.
	A + P	64.2 ± 0.4 ^c^	0.5 ± 0.0 ^a^	9.1 ± 0.1 ^f^	17.7 ± 0.1 ^f^
	AP	68.4 ± 0.0 ^d^	4.1 ± 0.2 ^c,d^	7.3 ± 0.3 ^c^	18.7 ± 0.7 ^f^
GB	RM	16.5 ± 0.6 ^A,B^	11.6 ± 0.2 ^B^	60.9 ± 0.1 ^A^	5.3 ± 0.0 ^B^
	A	70.0 ± 1.0 ^e^	5.6 ± 0.5 ^d^	4.5 ± 0.1 ^a^	16.5 ± 0.2 ^e^
	P	72.9 ± 0.5 ^f^	11.9 ± 1.0 ^f^	7.7 ± 0.0 ^d^	12.7 ± 0.0 ^b,c^
	A + P	59.6 ± 0.8 ^b^	12.1 ± 1.2 ^f^	7.2 ± 0.1 ^c^	17.7 ± 0.8 ^f^
	AP	77.1 ± 0.5 ^g^	9.3 ± 0.0 ^e^	5.5 ± 0.1 ^b^	9.7 ± 0.3 ^a^
NP	RM	19.3 ± 0.3 ^B^	9.1 ± 0.9 ^A^	72.9 ± 0.1 ^C^	2.4 ± 0.2 ^A^
	A	82.2 ± 0.2 ^h^	2.0 ± 0.4 ^a,b^	4.3 ± 0.1 ^a^	13.4 ± 0.3 ^c^
	P	86.5 ± 0.1 ^i^	n.d.	5.3 ± 0.0 ^b^	12.3 ± 0.1 ^b^
	A + P	68.6 ± 0.1 ^d^	3.6 ± 0.1 ^b,c^	8.7 ± 0.1 ^e^	16.3 ± 0.2 ^e^
	AP	81.7 ± 0.1 ^h^	3.3 ± 1.0 ^b,c^	5.2 ± 0.0 ^b^	12.7 ± 0.0 ^b,c^

n.d.—not determined

**Table 2:** Degree of hydrolysis (DH), hydrolysis yield (Yh), and protein yield (Yp) for the different raw materials hydrolysates with Alcalase 2.4L (A), Protana Prime (P), the sequential addition of these enzymes (A + P), and for simultaneous addition (AP). Different lowercase letters in each column indicate significant differences (*p* < 0.05) between FPH.

Hydrolysate Raw Material	Enzyme	DH (%)	Y_hydrolysis_ (%)	Y_protein_ (%)
		180 min/360 min		
BS	A	22.5 + 0.4 ^c,d^	12.5 ± 0.2 ^c^	68.3 ± 0.1 ^d^
	P	24.8 + 0.2 ^d,e^	n.d.	n.d.
	A + P	26.1 + 0.5 ^e^/57.9 + 0.8 ^l^	15.7 ± 0.3 ^d,e^	73.3 ± 0.4 ^f^
	AP	41.3 + 0.5 ^h^/48.5 + 0.9 ^j^	14.7 ± 0.3 ^c^	71.9 ± 0.0 ^e^
GB	A	23.2 + 0.6 ^d, e^	16.5 ± 0.3 ^d^	71.3 ± 1.0 ^e^
	P	19.7 + 1.1 ^b, c^	5.9 ± 0.1 ^b^	26.2 ± 0.2 ^b^
	A + P	18.7 + 0.3 ^b^/38.9 + 0.4 ^g,h^	18.8 ± 0.2 ^f^	67.9 ± 0.9 ^d^
	AP	37.7 + 2.8 ^g^/45.0 + 0.8 ^i^	16.9 ± 0.3 ^e^	80.7 ± 0.7 ^g^
NP	A	17.7 + 2.9 ^b^	14.0 ± 1.76 ^c^	55.4 ± 0.1 ^c^
	P	2.4 + 0.4 ^a^	3.4 ± 0.2 ^a^	16.2 ± 0.0 ^a^
	A + P	18.3 + 0.7 ^b^/45.2 + 1.8 ^i^	11.3 ± 0.3 ^g^	79.7 ± 0.1 ^g^
	AP	25.6 + 0.6 ^d,e^/34.1 + 1.5 ^f^	12.6 ± 0.4 ^f^	80.8 ± 0.1 ^g^

n.d.—not determined.

**Table 3:** Molecular weight distribution (percentage of total area) obtained for the hydrolysates from the different raw materials with Alcalase 2.4L (A) and Protana Prime (P) at the final stage (180 min, and with the sequential addition of these enzymes (Alcalase followed by Protana, A + P) and simultaneous addition (Alcalase and Protana, AP) at an initial stage (5 min), middle point (180 min), and final stage (360 min).

Enzyme	Time (min)	Range	Peak Area (%)		
			BS	GB	NP
Alcalase (A)	180	>1000 Da	23.6	19.7	21.3
		1000–500 Da	13.0	20.2	10.5
		500–100 Da	55.1	56.1	68.3
		<100 Da	8.2	4.0	---
Protana (P)	180	>1000 Da	n.d.	4.7	5.1
		1000–500 Da	n.d.	22.2	6.2
		500–100 Da	n.d.	68.9	83.6
		<100 Da	n.d.	4.3	5.04
Alcalase followed by Protana (A + P)	5	>1000 Da	26.3	17.7	17.2
		1000–500 Da	6.2	18.0	4.6
		500–100 Da	59.1	60.6	78.2
		<100 Da	8.3	3.8	---
	180	>1000 Da	24.1	15.4	21.8
		1000–500 Da	12.1	21.0	8.8
		500–100 Da	53.6	59.0	69.5
		<100 Da	10.2	4.6	---
	360	>1000 Da	15.8	7.67	9.0
		1000–500 Da	11.3	19.89	---
		500–100 Da	54.3	62.2	80.9
		<100 Da	18.6	10.23	10.1
Alcalase and Protana (AP)	5	>1000 Da	24.9	17.2	22.2
		1000–500 Da	5.0	16.8	4.9
		500–100 Da	59.2	62.8	68.8
		<100 Da	11.0	3.1	4.1
	180	>1000 Da	14.4	10.0	15.7
		1000–500 Da	17.5	17.4	10.8
		500–100 Da	50.2	60.6	66.5
		<100 Da	17.8	12.0	7.0
	360	>1000 Da	9.6	8.7	11.7
		1000–500 Da	25.3	16.1	---
		500–100 Da	47.2	62.1	79.3
		<100 Da	17.8	13.1	9.0

n.d.—not determined.

**Table 4:** Enzyme combinations and hydrolysis conditions (time, temperature, pH, and enzyme/substrate ratio).

Enzyme	Time (min)	Temperature (°C)	pH	E/S Ratio (*v/w*)
Alcalase (A)	180	60	8.5	1%
Protana (P)	180	55	5.5	1%
Alcalase + Protana (A + P)	180; 180	55	8.5 (A); 5.5 (P)	1% (A); 1% (P)
Alcalase and Protana (AP)	360	55	7	1% and 1%

## Data Availability

The original contributions presented in the study are included in the article/supplementary material, further inquiries can be directed to the corresponding author.

## Supplementary Materials

The following supporting information can be downloaded at: https://www.mdpi.com/article/10.3390/md23010014/s1, Figure S1: Diagram for processing co-products through various enzymatic treatments. Four different processes involving the enzymes Protana Prime (P), Alcalase 2.4L (A), Alcalase followed by Protana (A + P) and Alcalase and Protana added simultaneously (AP). Fish protein hydrolysates (FPH) were labeled as FPH P, FPH A, FPH A + P, and FPH AP, corresponding to the specific enzymatic treatment applied, resulting in a total of twelve FPH.
