# Supplementary material for: Enzymatic Hydrolysis Systems Enhance the Efficiency and Biological Properties of Hydrolysates from Frozen Fish Processing Co-Products
Source: Mar Drugs. 2024 Dec 28;23(1):14. doi: 10.3390/md23010014 (PMC11766955; doi:10.3390/md23010014)
Supplement: Supplementary file 1 [file marinedrugs-23-00014-s001.zip › marinedrugs-3367358-supplementary.pdf]

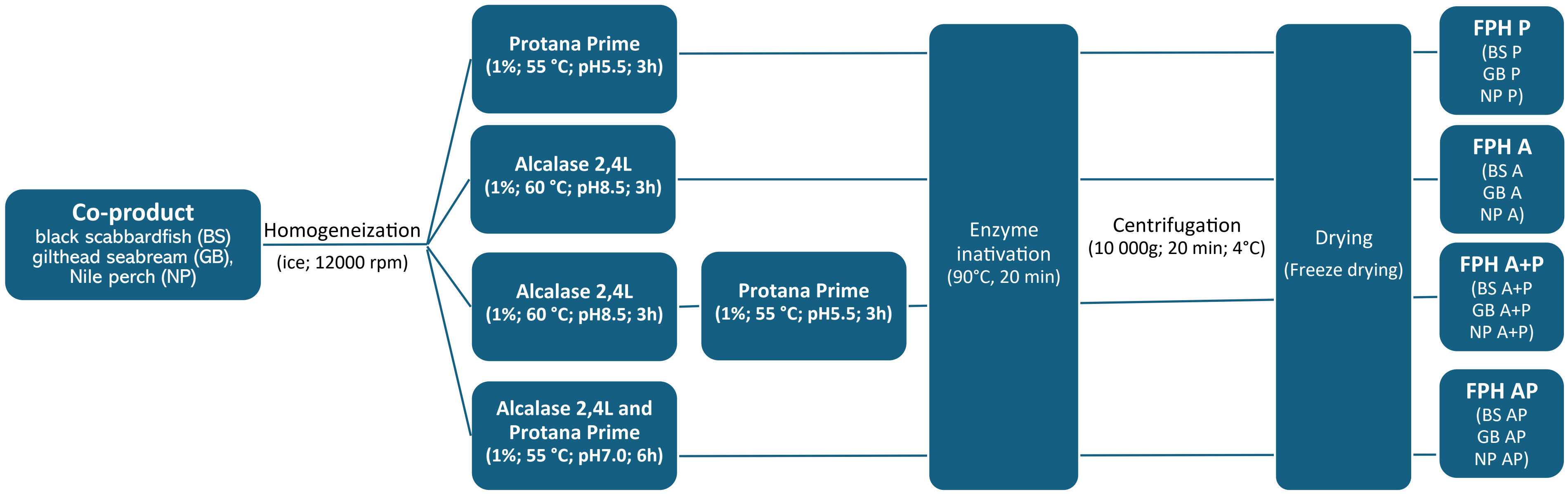

Figure S1 - Diagram for processing co-products through various enzymatic treatments. Four different processes involving the enzymes Protana Prime (P), Alcalase 2.4L (A), Alcalase followed by Protana (A + P) and Alcalase and Protana added simultaneously (AP). Fish protein hydrolysates (FPH) were labeled as FPH P, FPH A, FPH A+P, and FPH AP, corresponding to the specific enzymatic treatment applied, resulting in a total of twelve FPH.
